# Exosomal microRNAs in breast cancer: towards theranostic applications

**DOI:** 10.3389/fmolb.2024.1330144

**Published:** 2024-02-22

**Authors:** Richa Gulati, Tridip Mitra, Rohan Rajiv, Emilda Judith Ezhil Rajan, Chris Pierret, Elizabeth Ann L. Enninga, Rajiv Janardhanan

**Affiliations:** ^1^ Division of Medical Research, Faculty of Medicine and Health Sciences, SRM Institute of Science and Technology, Kattankulathur, Tamil Nadu, India; ^2^ Dietrich School of Arts and Sciences, University of Pittsburgh, Pittsburgh, PA, United States; ^3^ Department of Biochemistry and Molecular Biology, Mayo Clinic, Rochester, MN, United States; ^4^ Department of Obstetrics and Gynecology, Mayo Clinic, Rochester, MN, United States

**Keywords:** breast cancer, exosomal miRNAs, diagnostic markers, drug resistance, drug sensitivity

## Abstract

Breast cancer is one of the top two reproductive cancers responsible for high rates of morbidity and mortality among women globally. Despite the advancements in the treatment of breast cancer, its early diagnosis remains a challenge. Recent evidence indicates that despite the adroit use of numerous strategies to facilitate rapid and precision-oriented screening of breast cancer at the community level through the use of mammograms, Fine-needle aspiration cytology (FNAC) and biomarker tracking, no strategy has been unequivocally accepted as a gold standard for facilitating rapid screening for disease. This necessitates the need to identify novel strategies for the detection and triage of breast cancer lesions at higher rates of specificity, and sensitivity, whilst taking into account the epidemiologic and social-demographic features of the patients. Recent shreds of evidence indicate that exosomes could be a robust source of biomaterial for the rapid screening of breast cancer due to their high stability and their presence in body fluids. Increasing evidence indicates that the Exosomal microRNAs- play a significant role in modifying the tumour microenvironment of breast cancers, thereby potentially aiding in the proliferation, invasion and metastasis of breast cancer. In this review, we summarize the role of ExomiRs in the tumour microenvironment in breast cancer. These ExomiRs can also be used as candidate biomarkers for facilitating rapid screening and triaging of breast cancer patients for clinical intervention.

## Graphical Abstract

**Figure:**
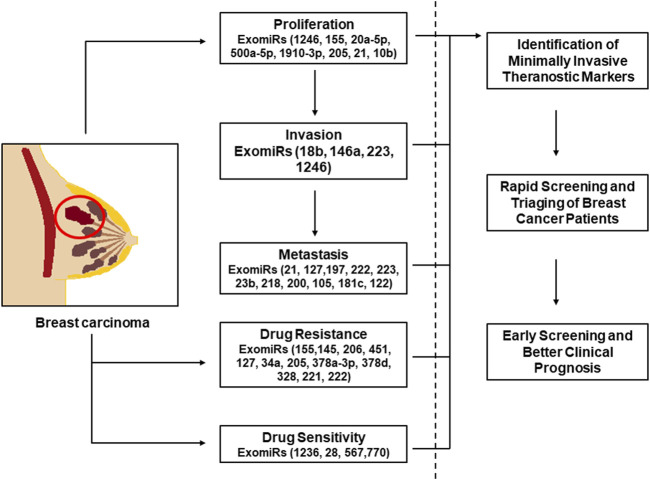
The graphical abstract shows the ExomiRs involved in the process of proliferation, metastasis and invasion of breast cancer along with the ExomiRs involved in the drug resistance and sensitivity. This implies the diagnostic as well as therapeutic potential of these ExomiRs with respect to breast cancer, indicating that these can be used a theranostic biomarkers for early diagnosis and treatment of breast cancer.

## 1 Introduction

Breast cancer is one of the most common cancers among women, with cases estimated to be 2.2 million in 2020 globally (Breast, 2020). Despite significant advances in the diagnosis and treatment of breast cancer, it remains one of the leading causes of morbidity among women. According to research, the 5-year survival rates for women with localized breast cancer, regional stage, and metastasis are 99 percent, 84 percent, and 23 percent, respectively (Desantis et al., 2012; Lowry et al., 2015). This suggests that early detection of the disease and treatment can lower breast cancer death rates. With a median overall survival of between 0.5–2.2 years, advanced-stage breast cancer is still incurable (Yeo et al., 2014). Histologically, breast cancer can be divided into invasive (ductal carcinoma *in situ*) and pre-invasive types (ductal carcinoma). Clinically significant classifications of the intrinsic, molecular subtypes of invasive breast carcinomas include luminal B-like HER2-positive, luminal B-like HER2-negative, and luminal A breast cancer, as well as triple-negative breast cancer (TNBC), HER2-enriched (nonluminal), and luminal B-like HER2-positive (Cheang et al., 2015; Nagini, 2017). Early detection of breast cancer, improving primary tumour treatment efficacy to prevent residual disease or metastatic seeding, and/or encouraging cell dormancy in metastatic niches are all the current strategies and roadblocks for reducing metastatic disease (Sempere et al., 2017).

Apart from women, breast cancer is also diagnosed among males, although it is a rare disease among them with an incidence rate of approximately 0.5%–1% (WHO, 2024). For male breast cancer, the median age of diagnosis is 68 years old, whilst for women it is 62 (Khan and Tirona, 2021). In terms of clinical presentation, histologic type distribution, and hormone-receptor expression, male breast cancer is distinct from female breast cancer (Gucalp et al., 2019; Fox et al., 2022). Male breast cancers usually appear clinically later and are primarily estrogen receptor (ERα) positive (up to 95%). Human epidermal growth factor receptor 2 (HER2, also known as ERBB2) expression is rare, and triple negativity is especially unusual in men (Chatterji et al., 2023). When compared to females with the same classification of the disease, males with breast cancer have a worse survival rate due to several factors that impact their prognosis (Ottini et al., 2010). There is a lack of studies exploring the mechanisms and risk factors involved in male breast cancer when compared to female breast cancer due to its low incidence and prevalence rates across the world. Researchers are working on identifying novel biomarkers for male breast cancer and have met some success, however, these biomarkers are not validated individually suggesting biomarkers for male breast cancer still remain under-investigated.

Extracellular vesicles (EVs) include a subtype called exosomes, which are coated in 30–100 nm lipid bilayers that contains a variety of bioactive substances such as lipids, proteins, nucleic acids, and small non-coding RNAs (Green et al., 2015; Wang M. et al., 2019). Almost all eukaryotic cells, including tumour cells and healthy stromal cells, have the ability to produce exosomes. Exosomes are released at higher concentrations by cancer cells compared to healthy proliferative cells (Sempere et al., 2017; Wang M. et al., 2019). Exosomes have crucial functions in intracellular and intercellular communication, including the elimination of toxic or damaged cell components and the stimulation of immune cells (Wang M. et al., 2019). Cancer-derived exosomes have been identified as key participants in the interaction between cancer cells and possible new microenvironments coined “pre-metastatic niches” because of their crucial involvement in cell-cell communication. Exosomes have recently piqued the interest of academics, primarily due to their potential as non-invasively available biomarkers, as they circulate in body fluids such as blood, urine, faeces, and saliva (Bahrami et al., 2018).

MicroRNAs (miRNAs) are endogenous noncoding RNAs that are short (21–23 nt) and have been demonstrated to regulate gene expression in a variety of physiological activities (O'Brien et al., 2018; Youngman and Claycomb, 2014). Numerous studies in recent years have demonstrated that different microRNAs have a role in direct or indirect interactions between breast cancer cells and tumour microenvironment (TME) components (Zhang et al., 2010; Challagundla et al., 2014). Although some of these studies hypothesized that miRNA had cell-extrinsic effects by regulating secreted factors (Pencheva and Tavazoie, 2013), the identification of miRNAs as a plentiful cargo in exosomes, called exosomal microRNAs (ExomiRs) (Graveel et al., 2015) raised the possibility of direct, miRNA-mediated regulation in recipient cells both close to and far from the donor cell expressing the miRNA(s). miRNAs have long been known to play a role in the diagnosis, onset, progression, prognosis, and response to treatment of breast cancer (Lu et al., 2005; Calin and Croce, 2006). Additionally, miRNAs are circulating in bodily fluids, and their expression in peripheral blood may serve as a biological marker for the prognosis and differential diagnosis of breast cancers (Kurozumi et al., 2017; Bahrami et al., 2018). Furthermore, several cell line studies show the effectiveness of ExomiRs in promoting both drug resistance as well as drug sensitivity. The ExomiRs that are generally upregulated in breast cancer cell lines are seen to be associated with an increased drug resistance to several drugs like doxorubicin, mitoxantrone, and tamoxifen, whereas the ExomiRs that are downregulated are seen to promote sensitivity to drugs like doxorubicin, cisplatin, and trastuzumab.

This review focuses on the need for prospecting ExomiRs as the potential theranostic markers to meet the unmet clinical need to both diagnoses as well as therapeutic treatment of breast cancer in women.

## 2 Diagnostic potential of exosomal microRNAs in breast cancer

**FIGURE 1:**
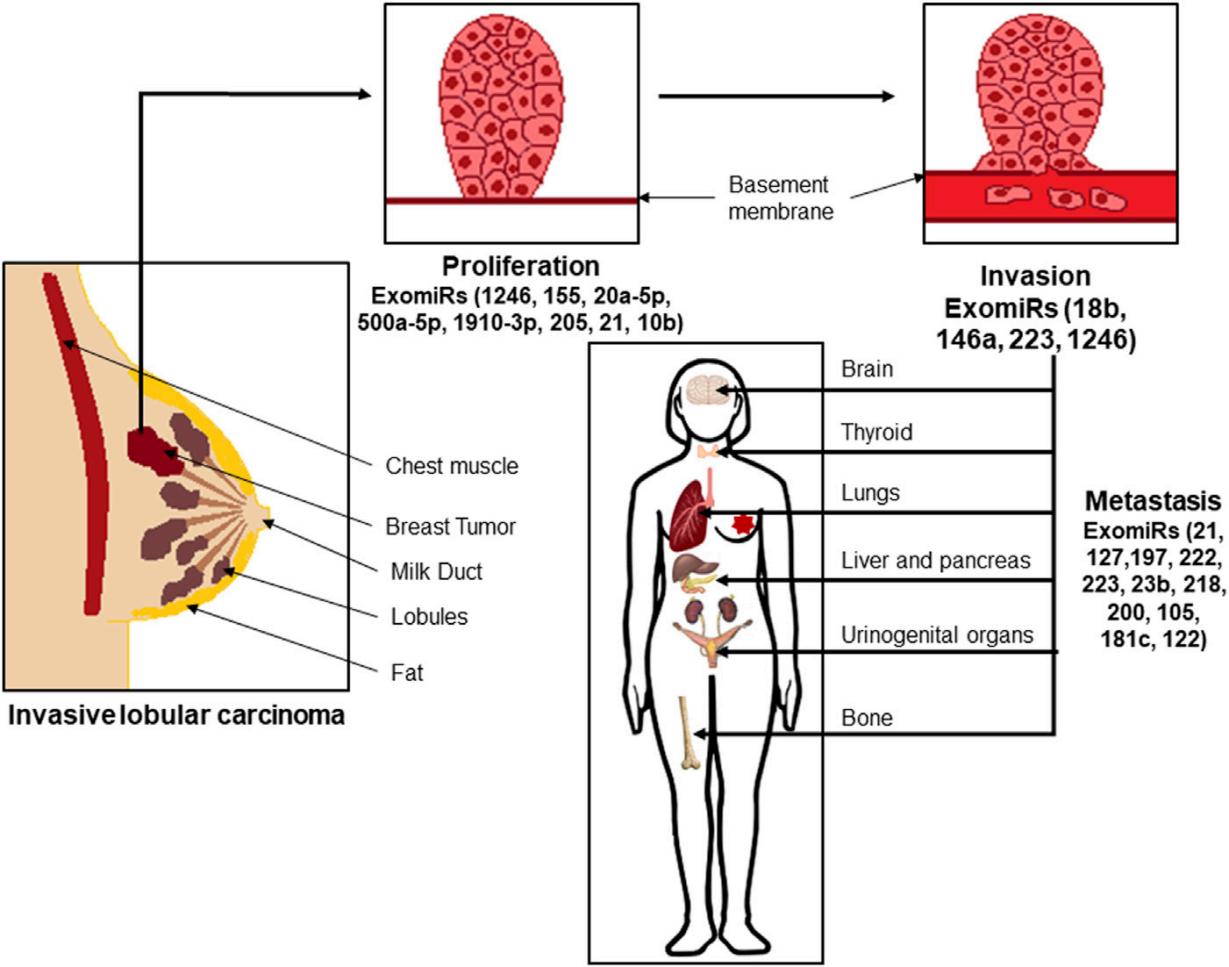
Exosomal microRNAs (ExoMiRs) play a critical role in the proliferation, invasion as well as metastasis of breast cancer. Such invasive lobular carcinomas are accompanied by the expression of different ExomiRs modulating the process of proliferation of the cells, followed by breaking the basement epithelial membrane leading to invasion and migration of the cells into the bloodstream, and metastasis to other organs like the brain, thyroid, lungs, etc.

Over recent years, it has been established that cancer patients have more ExomiRs than their healthy counterparts (Sempere et al., 2017; Wang M. et al., 2019). This shows that as opposed to exosomes formed from unaffected normal cells, ExomiRs in cancer patients more frequently reflect exosomes from cancer cells and/or other cell types in the tumour microenvironment (TME). Hence, detection/identification of these ExomiRs could help in better diagnosis or clinical prognosis of disease (Figure 1) (Table 1).

**TABLE 1:** Relationship of ExomiRs modulated by specific pathways in proliferation, invasion, and metastasis of breast cancer.

Exosomal miRs	Cell lines/Animal model	Target pathways/Proteins	Alteration mode	Reference
ExomiR-1246	MDA-MB-231, HMLE	CCNG2	Upregulated	Li et al. (2017), Li et al. (2018a)
ExomiR-155	MDA-MB-231, MCF-7, SKBR3	SOCS6, JAK2/STAT3	Upregulated	Jiang et al. (2010), Shen et al. (2015)
ExomiR-20a-5p	MDA-MB-231	SRCIN1	Upregulated	Guo et al. (2019)
ExomiR-500a-5p	CAF	USP28	Upregulated	Chen et al. (2021)
ExomiR-1910-3p	MCF-7, MDA-MB-231	MTMR3	Upregulated	Wang et al. (2020)
ExomiR-205	MCF-7/TAMR-1	E2F1	Upregulated	Zhao et al. (2021a)
ExomiR-21, ExomiR-10b	MCF10A	PTEN, HOXD10	Upregulated	Ma et al. (2007), Yan et al. (2011)
ExomiR-23b	Bone marrow-metastatic breast cancer cells	MARCKS	Downregulated	Ono et al. (2014)
ExomiR-218	Osteoblasts	COL1A1, YY1, and INHBB	Upregulated	Liu et al. (2018)
ExomiR-200	Murine breast cancer cells	ZEB2	Upregulated	Le et al. (2014)
ExomiR-181c	MDA-MB-231-luc-D3H2LN	PDPK1	Upregulated	Tomi et al. (2015)
ExomiR-122	TNBC	SLC2A1	Upregulated	Fong et al. (2015)
ExomiR-18b	MCF-7, MDA-MB-231	TCEAL7, NF-κB	Upregulated	Yan et al. (2021)
ExomiR-146a	MCF-7	Wnt signaling pathway	Upregulated	Yang et al. (2020)
ExomiR-223	Obese murine models	Mef2c	Upregulated	Li et al. (2022)

### 2.1 ExomiRs in breast cancer proliferation

Cancer cell growth and tumorigenesis are both affected by increased proliferation and decreased apoptosis. In comparison to microvesicles originating from healthy mammary epithelial cells, a study found that microvesicles from MDA-MB-231 breast cancer cells could provide a transformed-like phenotype to normal fibroblasts and epithelial cells, which is characterized by anchorage-independent proliferation and improved survival (Antonyak et al., 2011). Another study by Melo *et al.* showed that miRNA biogenesis in exosomes aids in the progression of the tumour (Melo et al., 2014). In a MDA-MB-231 cell line study, ExomiR-1246 was found to induce a tumour-promoting phenotype, including enhanced cell proliferation (Li X. J. et al., 2018). Furthermore, miR-1246 binds to the 3′UTR of Cyclin-G2(CCNG2) thereby directly targeting CCNG2 expression and leading to the downregulation in the expression of the tumour suppressor gene in non-malignant HMLE cells (Li X. J. et al., 2018). Ectopic ExomiR-155 overexpression boosted tumour proliferation in the breast cancer cell lines MDA-MB-231 and MCF-7 (Jiang et al., 2010). Another study involving ExomiR-155 showed that it inhibits SOCS6, activating the JAK2/STAT3 signaling pathway leading to the proliferation and differentiation of SKBR3 cells and MCF-7, which ultimately results in breast cancer cells becoming resistant to tamoxifen (Shen et al., 2015).

Exosomal miRNAs were shown to be much more abundant in metastatic breast cancer cells (MDA-MB-231 and 4T1) than in non-metastatic breast cancer cells or normal breast cells (MCF10A and NMuMG) (He et al., 2018). By inhibiting SRCIN1, a Src protein kinase involved in the control of cell migration, ExomiR-20a-5p could encourage proliferation and migration in Triple-negative breast cancer (TNBC) cells and accelerate osteoclastogenesis in mouse bone marrow-derived macrophages (Guo et al., 2019). A study by Chen *et. al.,* demonstrated that cancer-associated fibroblasts (CAF)-derived exosomes substantially aided tumour proliferation in breast cancer cell lines (Chen et al., 2021). By binding to ubiquitin-specific peptidase 28 (USP28), ExomiR-500a-5p was transported from CAFs to the cancer cells, where it promoted proliferation and metastasis (Chen et al., 2021).

Wang *et. al.,* showed that mammary epithelial cells and breast cancer cells exhibited proliferation and migration when ExomiR-1910-3p, which regulates myotubularin-related protein 3 (MTMR3), was overexpressed *in vitro* and *in vivo*, whereas miR-1910-3p silencing dramatically reduced these functions in both types of cells (Wang et al., 2020). ExomiR-205 can target E2F1 *in vivo* and *in vitro* to enhance tamoxifen resistance and proliferation in breast cancer (Zhao et al., 2021a). In MCF10A cells (normal breast cell line) treated with cancer exosomes, upregulation of miR-21 and miR-10b was seen (Ma et al., 2007; Yan et al., 2011). These two miRNAs have been linked to the progression of breast cancer. On the other hand, tumour suppressors PTEN and HOXD10, the known targets of miR-21 and miR-10b respectively, were found to be downregulated (Ma et al., 2007; Yan et al., 2011). It is important to understand that various ExomiRs involved in breast cancer population might vary among individuals of different origin as well as different breast cancer subtypes. An extensive research on these molecules is needed to better understand the diagnostic potential of the ExomiRs involved in the process of proliferation of tumour. Targeting these ExomiRs might provide an alternative approach for breast cancer intervention.

### 2.2 ExomiRs in breast cancer invasion and metastasis

The multistep process of metastasis includes the spread of tumour cells from the main tumour site, the development of a tumour microenvironment, and the colonization of distant organs by tumour cells (Turajlic and Swanton, 2016). Most deaths from breast cancer are the result of metastasis of the tumour, which highlights the significance of identifying novel biomarkers for metastasis for early diagnosis and effective treatment. In this section, we will focus on the ExomiRs involved in the metastasis of breast cancer.

Breast cancer has different subtypes, each having different characteristics with respect to mortality rate, aggressiveness, metastasis, and standard of intervention. Bone is the most preferred location for hormone receptor-positive (HR+) malignancies (Wu et al., 2017), where up to 70% of metastases originate (Chen et al., 2018). Although the methods by which primary breast tumours spread to the bone are still poorly understood, bone metastases have been found to reappear after 10years of remission, showing that metastatic breast cancer cells may remain dormant in the bone for long periods of time (Lim et al., 2011; Buonomo et al., 2017). According to a study by Yang et al., ExomiR-21 functions as exosomal cargo that is transported to prepare the bone metastatic location for breast cancer metastasis (Yang et al., 2020). Experiments revealed that by boosting osteoclast activity and bone lysis both *in vitro* and *in vivo*, exosomal miR-21 released by cancer cells serves as a crucial mediator for creating a favorable pre-metastatic environment. By targeting CXCL12, a stromal-cell-derived chemokine ligand, several ExomiRs (ExomiR-127, ExomiR-197, ExomiR-222, and ExomiR-223) contributed to a dormant phenotype in breast cancer bone metastases (Lim et al., 2011). Similarly, by inhibiting MARCKS, ExomiR-23b may promote the dormancy of bone marrow-metastatic breast cancer cells (Ono et al., 2014). ExomiR-218 can also influence the bone pre-metastatic niche by controlling the osteoblasts’ deposition of collagen through its targets COL1A1, YY1, and INHBB (Liu et al., 2018).

Most frequently connected with HR+ and Her2+ breast tumours, the liver is the second most frequent metastatic location after the brain, accounting for 40 to 50 percent of all cases (Zhao et al., 2018). Among all the subtypes of breast cancer, TNBC has the highest propensity to metastasize to the lungs first and then to other distant organs of the body (Van Mechelen et al., 2020). According to an *in vivo* study, ExomiR-200 contributed to the colonization of circulating murine breast cancer cells and promoted metastasis by transforming non-metastatic cells to metastatic by targeting ZEB2 (Le et al., 2014). An ExomiR-200 variation known as ExomiR-200c has also been linked to accelerated tumour development and subsequent lung metastasis over time (Zhang et al., 2017).

A higher metastatic potential and predictable brain metastasis are characteristics of aggressive breast carcinoma such as TNBC and HER-2 enriched (Wu et al., 2017). Patients with metastatic breast cancer are 10%–30% more likely to develop brain metastases, with breast cancer brain metastasis (BCBM) accounting for 15% of all cases (Ekici et al., 2016; Suh et al., 2020). A 2013 study suggested that nonmetastatic breast cancer cells could take up exosomes from brain metastasis, opening the door for exosomal functions in brain metastasis (Camacho et al., 2013). A study by Zhou *et. al.,* demonstrated that ExomiR-105 plays a significant role in the host’s vascular endothelial barrier destruction during the early stages of premetastatic niche development by specifically targeting cellular tight junctions (Zhou et al., 2014). Migration of breast cancer cells over the blood-brain barrier (BBB) is a critical step in the disease spreading to the brain. A study assessed how ExomiR-181c helps breast cancer cells pass through the BBB. By targeting PDPK1 in brain endothelial cells and disturbing the actin filament localization, ExomiR-181c translocation from breast cancer BC cells facilitates the breakdown of the BBB *in vitro* (Tomi et al., 2015). Exosomal miR-122 has been demonstrated to improve the Warburg Effect in premetastatic niches, particularly the brain and lungs, in TNBC cells. This study showed that increased levels of ExomiR-122 facilitate metastasis in breast cancer patients by increasing the availability of nutrients in the premetastatic niche (Fong et al., 2015).

Cancer cell invasion is the process where the tumour cells overcome the barriers of the extracellular matrix and spread into surrounding tissues (Krakhmal et al., 2015) followed by the formation of metastatic foci and metastasis (van Zijl et al., 2011). It is generally of two types, collective cell migration and individual cell migration, depending on the number of cancer cells participating in the invasive front (Krakhmal et al., 2015). This mesenchymal mechanism of invasion is a result of epithelial-mesenchymal transition (EMT) as the multicellular groups gain mesenchymal phenotypes and start to divide into single tumour cells by virtue of active dedifferentiation of a malignant epithelial tumour (Friedl et al., 2004). ExomiRs play a significant role in cell invasion and migration ultimately leading to metastasis (Li et al., 2022). ExomiR-18b is seen to induce tumour invasion and metastasis in mouse models as the overexpression of ExomiR-18b suppresses the expression of transcription elongation factor A-like 7 (TCEAL7) (Yan et al., 2021). TCEAL7 plays a key role in the reversing of Epithelial to Mesenchymal Transition (EMT) by suppression of the NF-κB pathway (Rattan et al., 2010). ExomiR-146a overexpression is also seen to regulate tumour invasion and migration by enhancing the Wnt signaling pathway in MCF-7 breast cancer cell lines (Yang et al., 2020). Overexpression of ExomiR-1246 is also seen to influence tumour cell proliferation and invasion in breast cancer cell lines as it suppresses the expression level of the target gene CCNG2 in MDA-MB-231 cells, an epithelial human breast cancer cell line (Li et al., 2017). The overexpression of ExomiR-223 also enhances migration and invasion of breast cancer by targeting the Mef2c gene thereby affecting the production of β-catenin in obese murine models (Li et al., 2022).

## 3 Therapeutic potential of exosomal microRNAs in breast cancer

miRNAs play critical roles in the progression of tumours; however, therapeutic miRNAs are making a run for FDA approval (Fire et al., 1998; Chakraborty et al., 2017). Several new biotech companies such as Miragen, MiRNA Therapeutics, and so on are trying to develop miRNA therapeutics however, drug delivery and resistance remain the biggest roadblocks in the field. MiRNAs can be packaged in liposomes, nanoparticles, and micelles similarly to conventional systemic drugs, but they have a limited ability to cross the blood-brain barrier (Krakhmal et al., 2015). However, no miRNA drug has yet to enter a phase III clinical trial because many phase II trials were stopped early due to serious adverse events, despite the fact that miRNAs can offer promising clinical applications (Austin, 2013; Chakraborty et al., 2017; Hanna et al., 2019).

**FIGURE 2:**
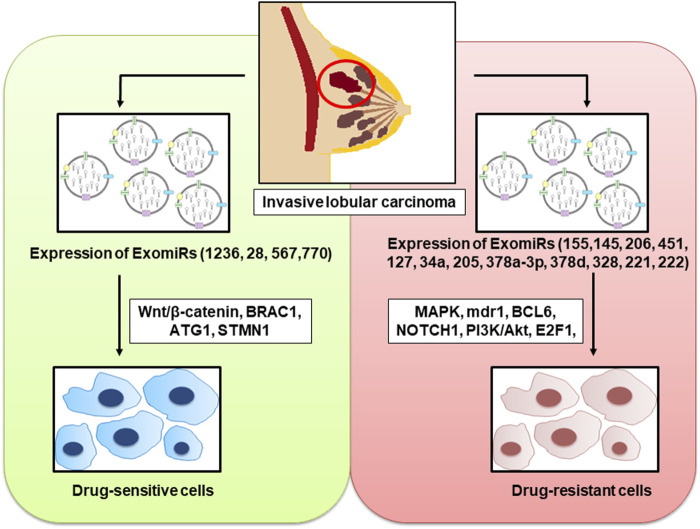
ExomiRs play a pivotal role in the incidence of both drug resistance and drug sensitivity in breast cancer leading to the alteration of several cellular metabolic pathways and proteins thereby influencing the progression, invasion, and metastasis of the carcinoma.

Most primary breast cancers initially respond well to miRNA treatment; however, show resistance at a later stage (Pondé et al., 2019). In order to overcome this resistance, ExomiRs and exosomes transporting other molecular cargo have been prospected as second-line treatment for breast cancer due to their significance in tumour drug resistance (Del Re et al., 2019) (Figure 2).

### 3.1 ExomiRs promoting drug resistance

An increase in the concentration of ExomiRs by several tumour cell types not only promotes tumour progression, angiogenesis, and metastasis but also plays a significant role in chemoresistance in all aggressive cancers, including breast cancer. This makes therapeutic treatments for breast cancer much more difficult (Cao et al., 2021). ExomiRs are highly stable molecules that are easily detectable in biological fluids like blood (Gallo et al., 2012), urine (Solé et al., 2019), and saliva (Gallo et al., 2012). Hence, they can act as potential cancer biomarkers useful both diagnostically and prognostically. These ExomiRs are seen to alter the expression of various proteins as they have strong binging affinity to the genes that are responsible for the expression of the protein thereby downregulating the protein expression in the body of the breast cancer patient, enhancing drug-resistance (Santos and Almeida, 2020). In breast cancer, miR-155 induces exosome-mediated chemoresistance to doxorubicin and paclitaxel, while simultaneously acting as an oncogenic signal reprogramming cancer metabolism (Santos et al., 2018; Ingenito et al., 2019). This resistance against cancer therapy is partially attributed to cancer progenitors as these cells arise from epithelial cells that undergo EMT (Santos et al., 2018). It was also found that the increase in chemoresistance was induced by the transfer of ExomiR-155 from resistant cancer cells to sensitive cells (Santos et al., 2018). Moreover, a study by Santos et al. shows the development of chemoresistance in MCF7 cell lines upon transfecting them with exosomal miRNA-155 mimics (Santos et al., 2018). ExomiR-155 is seen to produce doxorubicin resistance by inhibiting the MAPK signaling pathways in MCF7 breast cancer cell lines (Chen et al., 2010; Najminejad et al., 2019). The inhibition of the MAPK signaling pathway by ExomiR-155 also induces resistance to chemotherapeutic drug verapamil in MCF7 cell lines (Chen et al., 2010). A study by Chen et al. also showed that ExomiR-145 and ExomiR-206 interfere with the MAK signaling pathway in the MCF7 breast cancer cell line, leading to cross-resistance to daunorubicin and mitoxantrone (Chen et al., 2010). Overexpression of ExomiR-451 is also seen to induce doxorubicin resistance in MCF7 cell lines by inhibiting the expression of multidrug resistance R1 (mdr1) which in turn leads to pronounced downregulation of the Argonaute 2 protein, a member of the Argonaute protein family having an important role in RNA silencing (Kovalchuk et al., 2008). In the study by Kovalchuk et al., the overexpression of ExomiR-127 and ExomiR-34a was also seen to induce doxorubicin resistance (Najminejad et al., 2019). Western blot analysis shows that ExomiR-127 leads to an increase in the antiapoptotic protein BCL6 while ExomiR-34a increases the expression of the antiapoptotic protein NOTCH1 thereby inducing cell invasion and metastasis (Kovalchuk et al., 2008).

A study by Zhao, et al. suggests that ExomiR-205 induces tamoxifen resistance, thereby promoting proliferation, migration, and invasion and the suppression of apoptosis which were associated with activating the caspase pathway and phosphorylating Akt. The study also shows the E2F Transcription Factor 1 (E2F1) to be a direct target of ExomiR-205, in turn affecting the cellular transcription machinery in human breast cancer cells (Zhao et al., 2021b). *In vivo* studies suggest that intratumoral injection of ExomiR-205 to mice is correlated not only with a decrease in chemosensitivity but also with larger tumour size (Zhao et al., 2021b) as a result of horizontal transfer of these miRs (Chen et al., 2014). In breast cancer, chemotherapy activates the EZH2/STAT3 axis leading to the production of ExomiR-378a-3p and ExomiR-378d, resulting in chemotherapy-elicited, exosome-induced drug resistance (Yang et al., 2021). This is seen to occur by activating the Wnt and Notch stem cell signaling pathways via the targeting of Dickkopf-3 (a heat shock transcription factor 1 protein) and NUMB (an endocytic adaptor protein) expression in rodents (Pan et al., 2009). The overexpression of ExomiR-328 is seen to negatively regulate the expression of breast cancer resistance protein (BCRP/ABCG2) in human cancer cells leading to mitoxantrone resistance (Pan et al., 2009). It was also seen in a study by Miller et al. that the upregulation of ExomiR-221 and ExomiR-222 in MCF7 breast cancer cell line confers tamoxifen resistance by targeting p27Kip1, which induces caspase 7 and poly (ADP-ribose) polymerase cleavage, leading to its degradation, thereby activating MAPK and Akt signaling pathways favoring cell proliferation and survival (Miller et al., 2008) (Table 2).

**TABLE 2:** Relationship of ExomiRs with drug resistance modulated by specific pathways.

Exosomal miRs	Drugs	Target pathways/Proteins	Alteration mode	Reference
ExomiR- 155	Doxorubicin, Paclitaxel, Verapamil, Daunorubicin, Mitoxantrone	MAPK	Upregulated	Chen et al. (2010), Santos et al. (2018), Ingenito et al. (2019), Najminejad et al. (2019)
ExomiR- 145/206	Daunorubicin, Mitoxantrone	MAPK	Upregulated	Chen et al. (2010)
ExomiR- 451	Doxorubicin	MDR1	Upregulated	Kovalchuk et al. (2008)
ExomiR- 127	Doxorubicin	BCL6	Upregulated	Kovalchuk et al. (2008), Najminejad et al. (2019)
ExomiR- 34a	Doxorubicin	NOTCH1	Upregulated	Najminejad et al. (2019)
ExomiR- 205	Tamoxifen	PI3K/Akt, E2F1	Upregulated	Zhao et al. (2021b)
ExomiR- 378a-3p/378d	Multidrug resistance	Wnt/Notch	Upregulated	Pan et al. (2009)
ExomiR- 328	Mitoxantrone	BCRP/ABCG2	Upregulated	Pan et al. (2009)
ExomiR- 221/222	Tamoxifen	MAPK, PI3K/Akt	Upregulated	Miller et al. (2008)
ExomiR- 1236	Cisplatin	Wnt/β-catenin	Downregulated	Jia et al. (2020)
ExomiR-28	Doxorubicin	BRCA1	Downregulated	Kovalchuk et al. (2008)
ExomiR- 567	Trastuzumab	ATG5	Downregulated	Han et al. (2020)
ExomiR- 770	Doxorubicin	STMN1	Downregulated	Li et al. (2018b)

### 3.2 ExomiRs inhibiting drug resistance

Emerging evidence also suggests the possible role of certain ExomiRs in reducing chemoresistance in breast cancer cells. These ExomiRs are downregulated in breast cancer and both *in vitro* and *in vivo* evidence suggest their role in increasing the sensitivity of cells to the chemotherapeutic drugs, thereby not only decreasing the tumour size but also affecting the invasion, proliferation, and metastasis influencing the organotropism of breast cancer (Wong et al., 2020). ExomiR-1236 is seen to reduce cisplatin resistance by downregulating SLC9A1 and inactivating the Wnt/β-catenin pathway (Jia et al., 2020). Cisplatin is one of the most common chemotherapy regimens for several cancers including breast cancer, especially in the case of triple-negative breast cancers (Wang Y. et al., 2019). ExomiR-28 is seen to be downregulated in MCH7 breast cancer cell lines, leading to doxorubicin resistance (Kovalchuk et al., 2008). It was seen that upregulating ExomiR-28 leads to a loss of BRCA1 expression, which is associated with an increase in doxorubicin resistance in cell lines (Kovalchuk et al., 2008). Overexpression of ExomiR-567 in HER2-enriched cells was seen to directly target ATG5, thereby reversing the autophagy-dependent resistance to the targeted monoclonal antibody trastuzumab (Han et al., 2020). *In vivo* studies also suggest that overexpression of ExomiR-770 directly targets STMN1, a stathmin family phosphoprotein involved in intracellular signaling, thereby increasing doxorubicin sensitivity via inducing apoptosis and decreasing the tumour volume and metastasis (Li Y. et al., 2018) and (Table 2).

Taken together, ExomiRs not only act as an inducer of chemoresistance but also can aid as potential targets for endocrine therapies of breast cancer.

## 4 Conclusion

The strong regulatory effect of exosomal miRNA in the tumour microenvironment of breast cancer is evident and real. We are still in the early phase of research on exosomes in breast cancer, often encountering challenges despite the fact that this field has made considerable progress and certain sophisticated understandings have been attained. The ExomiRs mentioned in this review provides diagnostic as well as therapeutic potential against breast cancer. However, the combinatorial potential of these ExomiRs (theranostic potential) still remain an under-explored area in human beings. The studies that have identified ExomiRs with respect to breast cancer so far are majorly in cell lines/animal models, the very few ones in humans do not explore if they are niche-specific or are common for all populations. According to the statistical data by World Cancer Research Fund in 2020, the highest prevalence of breast cancer was recorded in European countries such as Belgium, Netherlands, Luxembourg, France, to name a few (WCRF, 2020), due to genetic reasons, lifestyle choices, as well as environmental factors. The world at large is known to have wide genetic diversity accompanied by divergent socio-cultural norms and lifestyle choices which are known to play a statutory role towards the creation of a highly heterogenous landscape of mutations associated with the onset, progression as well as clinical prognosis of breast cancer. The heterogeneity in the mutation landscape associated with breast cancer are expected to correspond to niche-specific variations in the ExomiRs whilst being prospected as theranostic markers for breast cancer. This niche-specific identification of ExomiRs will help in provisioning niche-specific theranostic regimens which cater to the needs of a specific community. Prospecting of ExomiRs as theranostic targets might be community-specific due to the extant variation in the genetic base of the populace. Hence, it might be imperative for the clinician researchers at large to take into account the perturbances at micro level which might influence the socio-cultural norms, diet and lifestyle choices of the populace in a niche-specific manner necessitating the need for big data with larger sample size having wide genetic base before prospecting the ExomiRs as theranostic targets.
