# Supplementary material for: Exosomal microRNAs in breast cancer: towards theranostic applications
Source: Front Mol Biosci. 2024 Feb 22;11:1330144. doi: 10.3389/fmolb.2024.1330144 (PMC10918471; doi:10.3389/fmolb.2024.1330144)
Supplement: Supplementary file 1 [file Table1.DOCX]

Table 1: Relationship of ExomiRs modulated by specific pathways in proliferation, invasion, and metastasis of breast cancer

| **Exosomal miRs** | **Cell lines/ Animal model** | **Target pathways/ Proteins** | **Alteration mode** | **Reference** |
| --- | --- | --- | --- | --- |
| ExomiR-1246 | MDA-MB-231, HMLE | CCNG2 | Upregulated | 29, 64 |
| ExomiR-155 | MDA-MB-231, MCF-7, SKBR3 | SOCS6, JAK2/STAT3. | Upregulated | 30, 31 |
| ExomiR-20a-5p | MDA-MB-231 | SRCIN1 | Upregulated | 33 |
| ExomiR-500a-5p | CAF | USP28 | Upregulated | 34 |
| ExomiR-1910-3p | MCF-7, MDA-MB-231 | MTMR3 | Upregulated | 35 |
| ExomiR-205 | MCF-7/TAMR-1 | E2F1 | Upregulated | 36 |
| ExomiR-21, ExomiR-10b | MCF10A | PTEN, HOXD10 | Upregulated | 37, 38 |
| ExomiR-23b | Bone marrow-metastatic breast cancer cells | MARCKS | Downregulated | 45 |
| ExomiR-218 | Osteoblasts | COL1A1, YY1, and INHBB | Upregulated | 46 |
| ExomiR-200 | Murine breast cancer cells | ZEB2, | Upregulated | 49 |
| ExomiR-181c | MDA-MB-231-luc-D3H2LN | PDPK1 | Upregulated | 55 |
| ExomiR-122 | TNBC | SLC2A1 | Upregulated | 56 |
| ExomiR-18b | MCF-7, MDA-MB-231 | TCEAL7, NF-κB | Upregulated | 61 |
| ExomiR-146a | MCF-7 | Wnt signaling pathway | Upregulated | 63 |
| ExomiR-223 | Obese murine models | Mef2c | Upregulated | 60 |
