# Supplementary material for: Exosomal microRNAs in breast cancer: towards theranostic applications
Source: Front Mol Biosci. 2024 Feb 22;11:1330144. doi: 10.3389/fmolb.2024.1330144 (PMC10918471; doi:10.3389/fmolb.2024.1330144)
Supplement: Supplementary file 2 [file Table2.DOCX]

Table 2: Relationship of ExomiRs with drug resistance modulated by specific pathways

| **Exosomal miRs** | **Drugs** | **Target pathways/Proteins** | **Alteration mode** | **Reference** |
| --- | --- | --- | --- | --- |
| ExomiR- 155 | Doxorubicin, Paclitaxel, Verapamil, Daunorubicin, Mitoxantrone | MAPK | Upregulated | 75, 76, 78, 79 |
| ExomiR- 145/206 | Daunorubicin, Mitoxantrone | MAPK | Upregulated | 79 |
| ExomiR- 451 | Doxorubicin | MDR1 | Upregulated | 80 |
| ExomiR- 127 | Doxorubicin | BCL6 | Upregulated | 78, 80 |
| ExomiR- 34a | Doxorubicin | NOTCH1 | Upregulated | 78 |
| ExomiR- 205 | Tamoxifen | PI3K/Akt, E2F1 | Upregulated | 81 |
| ExomiR- 378a-  3p/378d | Multidrug resistance | Wnt/Notch | Upregulated | 84 |
| ExomiR- 328 | Mitoxantrone | BCRP/ABCG2 | Upregulated | 84 |
| ExomiR- 221/222 | Tamoxifen | MAPK, PI3K/Akt | Upregulated | 85 |
| ExomiR- 1236 | Cisplatin | Wnt/β-catenin | Downregulated | 87 |
| ExomiR-28 | Doxorubicin | BRCA1 | Downregulated | 80 |
| ExomiR- 567 | Trastuzumab | ATG5 | Downregulated | 89 |
| ExomiR- 770 | Doxorubicin | STMN1 | Downregulated | 90 |
